# Multifaceted Mechanism of Amicoumacin A Inhibition of Bacterial Translation

**DOI:** 10.3389/fmicb.2021.618857

**Published:** 2021-02-12

**Authors:** Elena M. Maksimova, Daria S. Vinogradova, Ilya A. Osterman, Pavel S. Kasatsky, Oleg S. Nikonov, Pohl Milón, Olga A. Dontsova, Petr V. Sergiev, Alena Paleskava, Andrey L. Konevega

**Affiliations:** ^1^Petersburg Nuclear Physics Institute named by B. P. Konstantinov, NRC “Kurchatov Institute”, Gatchina, Russia; ^2^NanoTemper Technologies Rus, St. Petersburg, Russia; ^3^Center of Life Sciences, Skolkovo Institute of Science and Technology, Moscow, Russia; ^4^Department of Chemistry, Lomonosov Moscow State University, Moscow, Russia; ^5^Institute of Protein Research, Russian Academy of Sciences, Pushchino, Russia; ^6^Centre for Research and Innovation, Faculty of Health Sciences, Universidad Peruana de Ciencias Aplicadas (UPC), Lima, Peru; ^7^A. N. Belozersky Institute of Physico-Chemical Biology, Lomonosov Moscow State University, Moscow, Russia; ^8^Shemyakin-Ovchinnikov Institute of Bioorganic Chemistry, Moscow, Russia; ^9^Institute of Functional Genomics, Lomonosov Moscow State University, Moscow, Russia; ^10^National Research Centre “Kurchatov Institute”, Moscow, Russia

**Keywords:** amicoumacin A, antibiotic resistance, elongation factor EF-G, translocation, initiation, microscale thermophoresis, rapid kinetics

## Abstract

Amicoumacin A (Ami) halts bacterial growth by inhibiting the ribosome during translation. The Ami binding site locates in the vicinity of the E-site codon of mRNA. However, Ami does not clash with mRNA, rather stabilizes it, which is relatively unusual and implies a unique way of translation inhibition. In this work, we performed a kinetic and thermodynamic investigation of Ami influence on the main steps of polypeptide synthesis. We show that Ami reduces the rate of the functional canonical 70S initiation complex (IC) formation by 30-fold. Additionally, our results indicate that Ami promotes the formation of erroneous 30S ICs; however, IF3 prevents them from progressing towards translation initiation. During early elongation steps, Ami does not compromise EF-Tu-dependent A-site binding or peptide bond formation. On the other hand, Ami reduces the rate of peptidyl-tRNA movement from the A to the P site and significantly decreases the amount of the ribosomes capable of polypeptide synthesis. Our data indicate that Ami progressively decreases the activity of translating ribosomes that may appear to be the main inhibitory mechanism of Ami. Indeed, the use of EF-G mutants that confer resistance to Ami (G542V, G581A, or ins544V) leads to a complete restoration of the ribosome functionality. It is possible that the changes in translocation induced by EF-G mutants compensate for the activity loss caused by Ami.

## Introduction

Spread of antibiotic resistance in bacteria is one of the most urgent issues in medicine, well-known and long used antibiotics are losing their efficacy over time. The most promising ways to overcome this problem are creating analogs based on existing antibiotics, searching for novel natural substances with antibacterial activity, as well as thorough study of antibacterials and the resistance mechanisms to them (Nicolaou and Rigol, 2018; Witzky et al., 2019). Amicoumacin A (Ami) is a substance with the potential to become a new therapeutic antimicrobial agent. The molecular mechanism of action is not known in detail; however, preliminary investigations have suggested that Ami might have unusual mechanism of translation inhibition and bacterial cells might use previously undescribed resistance mechanism (Polikanov et al., 2014).

Ami was isolated for the first time in the early 1980s from marine Gram-positive bacteria *Bacillus pumilus* (Itoh et al., 1981). Later it was shown that other representatives of the genus *Bacillus* (Pinchuk et al., 2002), Gram-positive bacteria of the genus *Norcadia* (Sun et al., 2009), and Gram-negative *Xenorhabdus bovienii* (Park et al., 2016) could produce the antibiotic. Recent studies have identified clusters of the genes responsible for Ami synthesis and have demonstrated peculiar ways of silencing of Ami toxicity during its synthesis by producer strain cells (Li et al., 2015; Park et al., 2016; Terekhov et al., 2018). Ami belongs to a small group of 3,4-dihydroisocoumarin derivatives named AI-77 (Shimojima et al., 1982) structurally similar to xenocoumacins, bacilosarcins, and lipocoumacins (McInerney et al., 1991; Azumi et al., 2008; Li et al., 2012). The most famous representatives of amicoumacin group are amicoumacin A, B, and C varying in radicals at the C-12′ atom. It is considered that the presence of an amide group at this position ensures multiple effects of Ami (Hashimoto et al., 2007; Li et al., 2012). Indeed, Ami is a single representative of the group exhibiting anti-inflammatory, gastroprotective (Itoh et al., 1981), antitumor (Lama et al., 2012; Prokhorova et al., 2016), and strong antimicrobial activity against a wide range of microorganisms pathogenic to humans and animals (Pinchuk et al., 2001, 2002; Li et al., 2015; Gao et al., 2017; Terekhov et al., 2018).

**FIGURE 1:**
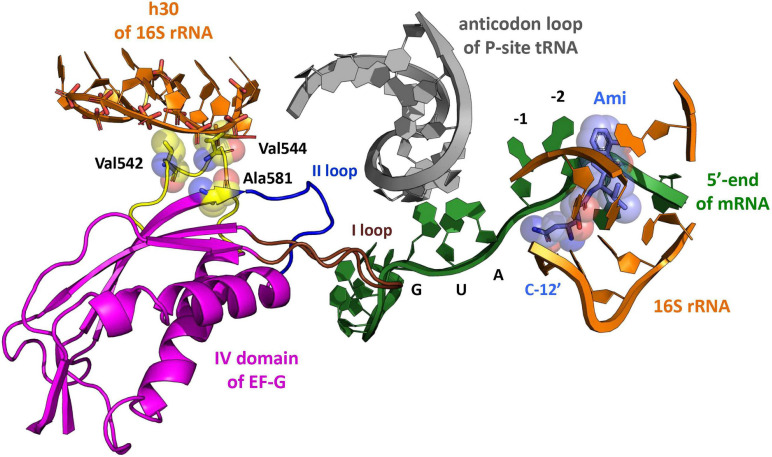
The model of Ami arrangement at the E site of the ribosome complex. The model presents Ami interaction (light blue) with the 5′-end of mRNA (green) and the implied interaction of the 16S rRNA (orange) with EF-G (magenta) amino acids (yellow), providing resistance to Ami. The EF-G conserved loops I and II are shown in brown and blue, respectively. The P-site tRNA is shown in light gray (PDB: 4V7D, 4V5F, 4W2F, and 4V9O).

Despite Ami was discovered a long time ago, little is known about its mechanism of action. It was recently shown that Ami activates several genes involved in various metabolic pathways (Lama et al., 2012), at the same time, the ribosome is considered to be its primary target for manifestation of antibacterial effect. Ami binds at the E site of the 30S subunit contacting conserved nucleotides U788, A794, C795 of the h24, nucleotide G693 of the h23, and nucleotides G1505 and U1506 of the h45 of the 16S rRNA, as well as the backbone of the mRNA at −1 and −2 nucleotides region without direct interaction with the E-site tRNA (Polikanov et al., 2014) (Figure 1). Ami binding site overlaps with those for other well-known antibiotics such as edeine, kasugamycin, and pactamycin (Pioletti et al., 2001; Schluenzen et al., 2006; Polikanov et al., 2014). Nevertheless, Ami utilizes a different way of interaction with mRNA and 16S rRNA molecules, namely, mRNA stabilization at the E site instead of displacing it, implying a unique way of translation inhibition (Polikanov et al., 2014). Initial studies of Ami showed that the main mode of action is aimed at translocation and to a lesser extent is associated with initiation (Polikanov et al., 2014; Prokhorova et al., 2016). However, the detailed inhibition mechanism by Ami has not yet been unveiled.

The unusual mechanism of translation inhibition by Ami forced bacterial cells to utilize novel previously undescribed resistance mechanism (Polikanov et al., 2014). Analysis of *Escherichia coli* and *Staphylococcus aureus* cells exhibiting resistance to Ami revealed specific mutations in genes coding for 16S rRNA, KsgA methyltransferase, and translation factor EF-G (Lama et al., 2012; Polikanov et al., 2014). Certain mutations in 16S rRNA and KsgA methyltransferase genes lead to resistance to pactamycin (Mankin, 1997) and kasugamycin (Helser et al., 1972; Schuwirth et al., 2006). In both cases the resistance mechanism was explained by the alteration of contacts between the antibiotic and the 30S subunit. Probably, C795U or A794G substitutions in the 16S rRNA, as well as deletion of 14 nucleotides (Δ424–437) in the *ksgA* gene or truncation of KsgA methyltransferase (L260Stp) lead to Ami resistance by the same mechanism. However, the antibiotic resistance due to amino acid substitutions in the domain IV of EF-G has not been described earlier. All found alterations G542V, G581A or ins544V in EF-G from *E. coli*, as well as G542V or G543S in EF-G from *S. aureus* (*E. coli* numbering) are located in the part of the factor that interacts with tRNA-mRNA complex on the ribosome (Gao et al., 2009), being at the same time more than 16 Å away from the binding site of Ami, suggesting a rather indirect mechanism of resistance (Polikanov et al., 2014) (Figure 1).

In this work, we perform a systematic investigation of Ami influence on the main steps of polypeptide synthesis and analyze the peculiarities of translocation catalyzed by variants of EF-G providing the resistance to Ami. The obtained results allow us to reveal the detailed mechanism of inhibition of bacterial translation by Ami.

## Materials and Methods

### Materials

The 30S and 50S subunits, 70S ribosomes, initiation factors (IF1, IF2, and IF3), fMet-tRNA^fMet^ (Milon et al., 2007), Phe-tRNA^Phe^, [^14^C]Phe-tRNA^Phe^, and [^14^C]Val-tRNA^Val^, EF-Tu (Wieden et al., 2002), yeast tRNA^Phe^(Prf16/17), tRNA^fMet^(Prf20) from *E. coli* (Wintermeyer et al., 1980), BPY-Met-tRNA^fMet^ (Holtkamp et al., 2014), and mRNA containing the sequence 5′-ACU ⋅ AUG ⋅ UUU-3′ and coding, respectively, Met-Phe- (Vinogradova et al., 2020) or containing 5′-ACU ⋅ AUG ⋅ GUU ⋅ UUU-3′ and coding Met-Val-Phe- were prepared according to the methods described previously. Ami was purified from *B. pumilus* INA 01087 strain as previously described (Polikanov et al., 2014). mRNA with UUC initiation codon was obtained by site-directed mutagenesis using primers 5′-GGTATACATACTTTCTTTACGATTACTACG-3′ and 5′-CGTAGTAATCGTAAAGATAAAGT-3′ on the basis of mRNA 5′-ACU ⋅ AUG ⋅ UUU-3′ and purified as previously described (Vinogradova et al., 2020).

The gene coding for an intact form (wt) or mutant variants (G542V, G581A, ins544V) of EF-G from *E. coli*, was cloned into the pCA24AN vector under IPTG-inducible T5 promoter (Polikanov et al., 2014). Intact or mutant forms of EF-G were expressed in the *E. coli* BL21 (DE3) strain and purified using affinity chromatography according to the previously published procedure (Cunha et al., 2013). All proteins contain 6× His-tag at the *N*-terminus.

All experiments were carried out in buffer TAKMx containing 50 mM Tris-HCl pH 7.5, 70 mM NH_4_Cl, 30 mM KCl, and X mM MgCl_2_ (7 mM ≤ X ≤ 21 mM).

### Complexes Formation

30S initiation complex (IC) was formed by incubation of 1 μM reactivated 30S subunits with 0.5 μM BPY-Met-tRNA^fMet^, 2 μM IF1, 1 μM IF2, 1.5 μM IF3, 0.5 mM GTP, and the corresponding mRNA in buffer TAKM_7_ for 30 min at 37°C. Before 30S IC formation, 30S subunits were reactivated by incubation in buffer TAKM_20_ for 30 min at 37°C.

70S IC was formed by incubation of 2 μM 70S ribosomes, 4 μM each of the initiation factors (IF1, IF2, and IF3), 12 μM mRNA coding Met-Phe-…, 4 μM initiator tRNA, 1 mM GTP, and 1 mM DTT in buffer TAKM_7_ for 1 h at 37°C. Where necessary, ICs were purified by gel-filtration chromatography on a BioSuite 450 HR SEC column, 7.8 × 300 mm (Waters, United States) in buffer TAKM_7_.

Ternary complex EF-Tu⋅GTP⋅Phe-tRNA^Phe^ was formed by incubation of 24 μM EF-Tu, 1 mM GTP, 1 mM DTT, 0.5 mg/ml pyruvate kinase (Roche Diagnostics, Switzerland), 3 mM phosphoenolpyruvate in buffer TAKM_7_ for 15 min at 37°C, then 12 μM Phe-tRNA^Phe^ was added and mixture was incubated for another 5 min at 37°C. Phe-tRNA^Phe^ was purified by HPLC and stored at −80°C, whereas Phe-tRNA^Phe^(Prf16/17) was prepared immediately before ternary complex formation. For this, 8 μM tRNA^Phe^(Prf16/17) was incubated with 0.2 mM Phe, 3 mM ATP, 6 μM 2-mercaptoethanol, 40 nM phenylalanine-tRNA synthetase, 40 nM tRNA nucleotidyltransferase in buffer TAKM_7_ for 30 min at 37°C.

Pretranslocation complexes were formed by mixing 70S initiation and ternary complexes with subsequent incubation for 1 min at 25°C. For purification, concentration of Mg^2+^ ions was increased to 21 mM, and the mixture of pretranslocation complexes was layered on 500 μl of the 1.1 M sucrose cushion (prepared in buffer TAKM_21_), followed by centrifugation in a SW55 rotor (Beckman Coulter, United States) at 55,000 rpm for 3 h at 4°C. The formed precipitate was dissolved in buffer TAKM_21_, shock frozen in liquid N_2_ and stored at −80°C.

### Microscale Thermophoresis

To determine the affinity of mRNA to 30S IC microscale thermophoresis method (MST) was used (Vinogradova et al., 2020). 30S IC was formed with BPY-Met-tRNA^fMet^ (initiator tRNA labelled with Bodipy FL at methionine moiety) and increasing concentrations of corresponding mRNA in buffer TAKM_7_ for 30 min at 37°C in the presence of 60 μM amicoumacin A, 30 μM edeine, 60 μM kasugamycin or without antibiotic. Detection of fluorescence changes was carried out on a Monolith NT.115 instrument (NanoTemper Technologies GmbH, Germany), using standard capillaries (Cat. MO-K022, Nanotemper Technologies, Germany). A green filter was applied, the power of monochromatic LED was 30%, and the intensity of IR laser was 40%. Each experiment was done in three replicates.

### Rapid Kinetics Experiments

Rapid kinetics were measured using a SX-20 stopped-flow apparatus (Applied Photophysics, Leatherhead, United Kingdom). Proflavin fluorescence was excited at 460 nm, Bodipy FL fluorescence was excited at 470 nm and measured after passing a cut-off filter KV490 nm (Schott, Mainz, Germany) in both cases. Light scattering experiments were carried out at 430 nm, followed by detection at 90° angle, without cut-off filter. Experiments were performed in buffer TAKM_7_, with 1 mM GTP and 1 mM DTT at 20°C (binding of aminoacyl-tRNA to the A site) (Wieden et al., 2002; Pan et al., 2008), 25°C (formation of the 70S IC) (Vinogradova et al., 2020) or 37°C (translocation) (Holtkamp et al., 2014). Where necessary, 30 μM amicoumacin A, 30 μM edeine, 60 μM kasugamycin, 30 μM tetracycline, 150 μM kirromycin or 200 μM viomycin were added. Samples were rapidly mixed in equal volumes. Time courses depicted in the figures were obtained by averaging 5–7 individual transients. Data were evaluated by fitting to a single-exponential function with a characteristic time constant (*k*_*app*_), amplitude (*A*), and final signal amplitude (*F*_0_) according to equation *F* = *F*_0_ + *A* × exp(−*k*_*app*_ × *t*) where *F* is the fluorescence at time *t*. Where necessary, two exponential terms were used with two characteristic time constants (*k_*app*1_*, *k_*app*2_*), amplitudes (*A*_1_, *A*_2_), according to equation *F* = *F*_0_ + *A*_1_ × exp(−*k*_*app*__1_ × *t*) + A_2_ × exp(−*k*_*app*__2_ × *t*). A hyperbolic function was used to analyze the concentration dependence of the translocation apparent rate constants from the concentration of elongation factor EF-G. Calculations were performed using Prism 6.02 software (GraphPad Software, United States). Standard deviations were calculated using the same software.

To study accommodation of initiator BPY-Met-tRNA^fMet^ and 50S subunit joining at the 70S IC formation, 0.1 μM 30S IC programmed with an appropriate mRNA were rapidly mixed with 0.3 μM 50S subunits (final concentrations after mixing are given throughout).

To study aminoacyl-tRNA binding to the A site of the ribosome, IC containing fMet-tRNA^fMet^ (Prf20) (initiator tRNA labelled with proflavine at position 20 in the elbow region of tRNA) was rapidly mixed with ternary complex. fMet-tRNA^fMet^(Prf20) was obtained immediately before the IC formation, as described previously (Milon et al., 2007).

To study the pre-steady-state kinetics of translocation, we used pretranslocation complexes containing deacylated tRNA^fMet^ at the P site and fluorescently labelled fMet-Phe-tRNA^Phe^ at the A site. To characterize movement of central part of tRNA upon translocation, we have used proflavine attached to dihydrouridine at position 16 and/or 17 located in the elbow region of fMet-Phe-tRNA^Phe^ [fMet-Phe-tRNA^Phe^(Prf16/17)] (Savelsbergh et al., 2000). To monitor displacement of the acceptor end of tRNA from the A to the P site we have used Met-Phe-tRNA^Phe^ labelled with Bodipy FL at methionine moiety (BPY-Met-Phe-tRNA^Phe^) (Holtkamp et al., 2014). Pretranslocation complexes were rapidly mixed with increasing concentration of wt or mutant variants of EF-G in the presence of 1 mM GTP.

### Peptide Bond Formation

To analyze formation of dipeptide fMet-Phe, 0.5 μM 70S IC containing fMet-tRNA^fMet^ at the P site were mixed with equal volume of 1 μM ternary complex EF-Tu⋅GTP⋅[^14^C]Phe-tRNA^Phe^ and incubated in buffer TAKM_7_, for 1 min at 25°C. Then samples were quenched with 1/10 volume of 5 M KOH and hydrolyzed for 30 min at 37°C. Samples were neutralized with 1/10 volume of glacial acetic acid and analyzed by reversed-phase HPLC. Percentage of synthesized dipeptide was determined by incorporation of the radioactive label, as described earlier (Rodnina and Wintermeyer, 1995). Experiments were done in four replicates.

To analyze formation of tripeptide fMet-Val-Phe, pretranslocation complexes, containing deacylated tRNA^fMet^ at the P site and fMet-[^14^C]Val-tRNA^Val^ at the A site, were formed and purified by centrifugation through sucrose cushion as described above. Then, a mixture of ternary complex EF-Tu⋅GTP⋅Phe-tRNA^Phe^ (1.6 μM) with EF-G wt or EF-G G542V (5 μM) was added to pretranslocation complexes (0.4 μM) in a quench-flow instrument KintTek RQF-3 (KinTek Corporation, United States). The experiments were performed in buffer TAKM_7_ with addition of 1 mM DTT and 1 mM GTP at 20°C. The reaction was stopped at certain time points by addition of 0.8 M KOH, and subsequent procedures for measurement of synthesized tripeptide fMet-[^14^C]Val-Phe were performed as described above for dipeptide formation analysis. The reaction rate constants were calculated using one or two exponential equations.

For the analysis of dipeptide formation, ICs were formed in the presence of 30 μM amicoumacin A or 2 μM madumycin II; for the analysis of tripeptide formation, purified pretranslocation complexes were preliminarily incubated with 30 μM amicoumacin A for 10 min at 25°C, and then mixed with other reaction components.

### Multiple Turnover Translocation

The reaction was performed according to previously published method (Savelsbergh et al., 2000). For experiments, 0.2 μM pretranslocation complexes containing deacylated tRNA^fMet^ at the P site and fMet-[^14^C]Val-tRNA^Val^ or fMet-[^14^C]Phe-tRNA^Phe^ at the A site, were purified by gel-filtration chromatography, and 3 nM wt or mutant form of EF-G was added. The reaction was carried out in buffer TAKM_14_ at 25°C. Where necessary, 30 μM amicoumacin A was added. For fMet-[^14^C]Val-Pmn or fMet-[^14^C]Phe-Pmn formation, posttranslocation complexes were mixed with 1 mM Pmn for 10 s at 37°C. The reaction was quenched by 1.5 M sodium acetate saturated with MgSO_4_. fMet-[^14^C]Val-Pmn or fMet-[^14^C]Phe-Pmn was extracted with ethyl acetate and quantified by radioactivity counting.

### Stability of Peptidyl-tRNA Binding at the A Site

Experiments and calculations were carried out according to previously published method (Konevega et al., 2004), namely: pretranslocation complexes (0.25 μM) containing deacylated tRNA^fMet^ at the P site and fMet-[^14^C]Val-tRNA^Val^ at the A site were purified by centrifugation through a sucrose cushion. To induce the dissociation of fMet-[^14^C]Val-tRNA^Val^ from the A site, the Mg^2+^ concentration in buffer TAKM was adjusted to 7, 8.5, 10, 15, or 20 mM, and the amount of pept-tRNA bound to the A site at different incubation times at 37°C (with or without 30 μM amicoumacin A) was determined by nitrocellulose filtration. Dissociation and association elemental rate constants, *k*_*off*_ and *k*_*on*_, were calculated from time courses of dissociation by numerical integration (Konevega et al., 2004). The following kinetic model was used: A ⇔ B + C, and B ⇒ D, where A denotes ribosomes with pept-tRNA bound to the A site; B, unbound pept-tRNA; C, ribosomes with unoccupied A site; D, hydrolyzed pept-tRNA. *k*_*hydr*_ is the rate constant of pept-tRNA hydrolysis free in solution. Equilibrium dissociation constant was calculated as *K*_D_ = *k_*off*_/k_*on*_*. Free energy (ΔG^0^) of binding was calculated from the *K*_d_ values according to the equation ΔG^0^ = -RTln(*K*_d_).

### nanoDSF

To detect conformational stability and identity of intact and mutant forms of EF-G nanoDSF method of Prometheus NT.48 (NanoTemper Technologies GmbH, Germany) was used. The melting point (*T*_m_, °C), temperature at which half of a protein was unfolded, was determined by the fluorescence intensity measurements of aromatic amino acid residues at 330, 350, and 350/330 nm ratio. Proteins were diluted to a final concentration of 5 μM in buffer TAKM_7_ in the presence of 1 mM GTP. For the reaction, 10 μl of the sample was taken using standard capillaries (Cat. PR-C002, Nanotemper Technologies, Germany). The melting experiment was performed with a step of 0.1°C/min in the temperature range 15–95°C, the laser intensity was 30%.

## Results

### Amicoumacin A Retards Initiation

Polypeptide synthesis starts with the initiation step that aims at correct positioning of an initiator fMet-tRNA^fMet^ over an mRNA start codon at the ribosomal P site. In bacteria this process is orchestrated by three protein initiation factors. IF1 associates with the A site of the small 30S ribosomal subunit and prevents aminoacyl-tRNA from entering. IF2 recognizes the *N*-formylmethionine moiety of an initiator fMet-tRNA^fMet^ and facilitates binding of this tRNA. IF3 takes part in the selection of an initiator tRNA, monitoring correct codon–anticodon interaction, and the 50S subunit joining, ensuring fidelity of IC formation. Initiation includes three main phases: 1) assembly of initiation factors, mRNA, and initiator fMet-tRNA^fMet^ on the 30S subunit into a pre-IC complex (30S pre-IC), 2) conversion of the 30S pre-IC into a stable 30S IC upon recognition of the mRNA start codon by the fMet-tRNA^fMet^, 3) joining of the 50S subunit to the 30S IC resulting in the 70S IC formation (Gualerzi and Pon, 2015). As amicoumacin A (Ami) contacts mRNA near the start codon region (Polikanov et al., 2014), it might influence mRNA binding to the 30S subunit and codon-anticodon recognition at the P site. In addition, one could expect impairment in joining of the 50S subunit to the 30S IC due to controlling function of IF3. To test this hypothesis, we compared the affinity of mRNA to the 30S IC, the ability of 30S IC to form 70S IC and to accommodate P-site tRNA in the presence of Ami and in antibiotic-free system taking into account IF3 participation.

#### Initiation in a Complete System

**FIGURE 2:**
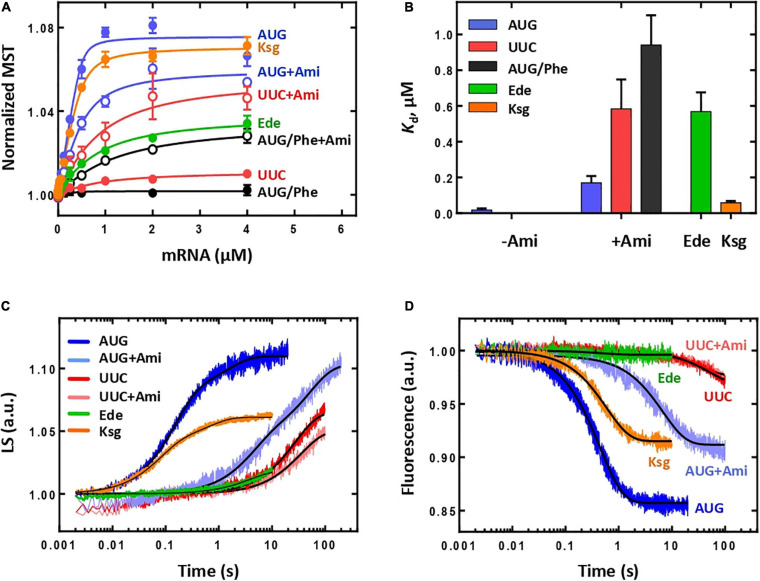
The influence of Ami on the initiation in a complete system. **(A)** Titration of the 30S IC formation with mRNA containing AUG start codon (denoted as AUG, in the presence of Ami, AUG + Ami; Ede, edeine; Ksg, kasugamycin), UUC start codon (denoted as UUC, in the presence of Ami – UUC + Ami), AUG start codon with the participation of the elongator BPY-Phe-tRNA^Phe^ (denoted as AUG/Phe, in the presence of Ami – AUG/Phe + Ami). **(B)** Dissociation constants (*K*_d_) calculated from **(A)**. The *K*_d_ value for mRNA with AUG in the absence of Ami is very low and practically not visible on the graph; the *K*_d_ values for mRNA with UUC start codon or AUG codon with the participation of Phe-tRNA^Phe^ in the absence of Ami are not shown as they do not form the initiation complex. **(C)** Association of the 50S subunit (0.3 μM) with the 30S IC (0.1 μM) monitored by light scattering. Time curves are labelled according to the mRNA start codon and presence of Ami, Ede, or Ksg. **(D)** Association of the 50S subunit (0.3 μM) with the 30S IC (0.1 μM) monitored by fluorescent signal from the BPY reporter of the initiator BPY-Met-tRNA^fMet^, labelling as in **(C)**.

We used the MST to monitor 30S IC formation by the fluorescence change of BPY-Met-tRNA^fMet^ (Vinogradova et al., 2020) at increasing concentrations of model mRNA (Calogero et al., 1988) (Figure 2A). The presence of Ami led to almost an order of magnitude decrease of the mRNA affinity [*K*_d_(without Ami) = 18 ± 9 nM; *K*_d_(with Ami) = 169 ± 39 nM], whereas the amplitude was reduced only by 20% (Figures 2A,B), suggesting that Ami retained the ability of the IC to be formed albeit with lower affinity for the mRNA. Formation of non-canonical 30S IC either with classical mRNA start codon AUG substituted to UUC or with initiator fMet-tRNA^fMet^ replaced by elongator Phe-tRNA^Phe^ was shown to be impaired (Figure 2A) (Vinogradova et al., 2020). However, addition of Ami stimulated the formation of non-canonical 30S IC. The binding values of the resulting complexes were in submicromolar range [*K*_d_(UUC + Ami) = 584 ± 165 nM; *K*_d_(AUG/Phe + Ami) = 940 ± 166 nM] (Figures 2A,B). The extent of 30S IC formation, as judged by the signal amplitude of the titration curves, was noticeably lower than that for the complexes with AUG start codon and initiator fMet-tRNA^fMet^, yet much higher if compared to the absence of Ami. Thus, Ami appears to reduce the affinity of optimal mRNAs but greatly enhances erroneous 30S complexes, highlighting the stabilizing effect of Ami for non-canonical initiation with an incorrect start codon or elongator tRNA. For additional validation of our results, we used two inhibitors of initiation that share binding site with Ami, edeine (Ede) and kasugamycin (Ksg). Ede inhibits tRNA binding to the P site by preventing codon–anticodon interaction (Dinos et al., 2004). Consistent with previous findings, MST assay revealed that 30S IC formation was greatly impaired by Ede. Only 52% of complexes were able to form as indicated by decrease of the titration curve amplitude. In addition, mRNA binding affinity to 30S IC was reduced more than 30 times [*K*_d_ (Ede) = 568 ± 108 nM], implying that Ede not only violates codon-anticodon base pairing but also interferes with mRNA binding. Ksg distorts the mRNA path in the ribosome leading to hypothesis of preventing recognition of the start codon by the initiator tRNA (Schluenzen et al., 2006; Schuwirth et al., 2006). However, the discovery of context-specific action of Ksg suggests that this antibiotic differentially inhibits initiation of translation of mRNAs depending on the sequence of the E-site and P-site codons (Chin et al., 1993; Vázquez-Laslop and Mankin, 2018). In our experiments, the presence of Ksg did not influence 30S IC formation. The amplitude of the titration curve and affinity [*K*_d_ (Ksg) = 59 ± 10 nM] were comparable with those obtained for canonical 30S IC (Figures 2A,B).

To verify whether Ami-containing 30S IC can form 70S ICs, we monitored joining of the 50S subunit to the 30S IC by the light scattering method in a stopped-flow apparatus. The increase of light scattering signal was approximated by two-exponential curve suggesting that the reaction had two phases, where the first phase had more than 70% of the amplitude signal with the apparent rate constant *k_*app*_* = 6.9 ± 0.1 s^–1^ (Figure 2C), in accordance to previous observations for the mRNA with the AUG start codon (Milon et al., 2008; Goyal et al., 2015). In the presence of Ede, no 70S IC formation was detected supporting strong influence of this antibiotic on 30S IC formation. Although Ksg did not influence 30S IC formation, it exhibited its action on the next step resulting in lower amount of associated subunits depicted by two times decrease of the signal amplitude. Ami has revealed different inhibiting mode: the total signal amplitude remained unchanged, whereas the first phase was two times smaller and the rate reduction was 30-fold (*k_*app*_* = 0.246 ± 0.008 s^–1^) (Figure 2C). Furthermore, we monitored how Ami affected the accommodation of the fluorescently labelled initiator BPY-Met-tRNA^fMet^ at the P site upon 50S subunit joining, as a late step of the 70S IC formation (Vinogradova et al., 2020). The decrease of the fluorescence signal corresponded to one-phase reaction (Figure 2D). Similarly to the subunit association step, Ami greatly delayed tRNA accommodation. The presence of the antibiotic reduced the amplitude of the signal to 57% at a 15-fold rate reduction [*k*_*app*_(without Ami) = 2.26 ± 0.02 s^–1^; *k*_*app*_(Ami) = 0.148 ± 0.002 s^–1^]. As it was expected from the previous initiation experiments, in the presence of Ede no tRNA accommodation was detected, whereas Ksg demonstrated only moderate inhibiting effect with two-fold decrease of the signal amplitude without significant influence on the rate of the reaction [*k*_*app*_(Ksg) = 1.70 ± 0.02 s^–1^]. The ICs containing mRNA with UUC start codon, regardless of the presence of Ami, demonstrated very low amplitude of fluorescence change (Figures 2C,D). The significant decrease of the reaction rate at both 50S subunit joining and the tRNA accommodation indicates the fact that erroneous 70S ICs are rarely formed.

To summarize, three initiation inhibitors sharing overlapping binding sites on the ribosome perform differently. Ede significantly impairs formation of 30S IC, most probably, due to displacement of mRNA preventing codon–anticodon interaction in the P site. Ksg, in contrast, does not affect 30S initiation and only modestly violates 70S IC formation. As Ksg is a context-specific antibiotic, its mild inhibiting effect could be associated with mRNA sequence used in the study. In the presence of Ami, 30S IC programmed with the mRNA with the classical AUG start codon forms 70S IC, albeit at slower rates. At the same time, our data show that Ami enhances formation of erroneous 30S ICs with the UUC start codon; however, these complexes are not able to build 70S ICs, likely because of IF3 kinetically checking the transition to elongation (Milon et al., 2008).

#### Initiation in a System Lacking IF3

**FIGURE 3:**
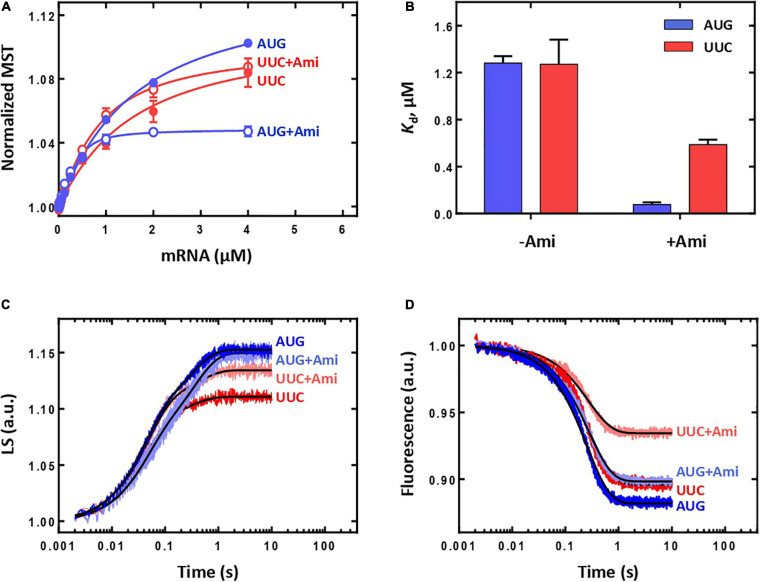
The influence of Ami on the initiation in a system lacking IF3. **(A)** Titration of the 30S IC formation in the absence of IF3 with mRNA containing either AUG or UUC start codon. **(B)** Dissociation constants (*K*_d_) calculated from **(A)**. **(C)** Association of the 50S subunit (0.3 μM) with the 30S IC in the absence of IF3 (0.1 μM) monitored by light scattering. **(D)** Association of the 50S subunit (0.3 μM) with the 30S IC in the absence of IF3 (0.1 μM) monitored by fluorescent signal from the BPY reporter of the initiator BPY-Met-tRNA^fMet^.

As IF3 plays a particularly important role in the fidelity of selection of initiator tRNA^fMet^ and correct start codon (Milon and Rodnina, 2012), we decided to study the involvement of IF3 in the mechanism of Ami-dependent inhibition of translation initiation. We assembled 30S IC in the absence of IF3 and examined the affinity of the mRNA with different start codons to such complexes. Although the affinity of the mRNAs for 30S IC lacking IF3 was very low [*K*_d_(AUG) = 1,282 ± 58 nM, *K*_d_(UUC) = 1,271 ± 210 nM], the titration curves amplitudes were rather large indicating that the complexes were formed (Figures 3A,B). The addition of the antibiotic led to significant increase in the mRNA affinity [*K*_d_(AUG + Ami) = 77 ± 18 nM, *K*_d_(UUC + Ami) = 587 ± 42 nM], making the values comparable with those obtained for the 30S IC formed with all components of the translation initiation. Thus, Ami increases the mRNA affinity for 30S complexes lacking IF3 regardless of the initiation codon at the P site.

We also analyzed the ability of the 30S IC lacking IF3 to proceed to the 70S IC. As expected, the absence of the factor increases the reaction rate of 50S subunit joining regardless of the initiation codon (Figure 3C) (Milon et al., 2008). The reaction rates for IC formation with mRNA with AUG or UUC start codons were practically the same [*k*_*app*_(AUG) = 25.6 ± 0.3 s^–1^; *k*_*app*_(UUC) = 22.9 ± 0.8 s^–1^] with 30% drop of signal amplitude for UUC codon at the P site. The presence of the antibiotic did not substantially contribute to the rate or amplitude of the reaction [*k*_*app*_(AUG + Ami) = 24.7 ± 0.7 s^–1^; *k*_*app*_(UUC + Ami) = 22.0 ± 0.7 s^–1^]. Thus, the mechanism of Ami inhibition during 70S IC formation appears to be IF3-dependent. The 30-fold rate reduction provoked by Ami (Figure 2C) is abolished in the absence of IF3 (Figure 3C). Regardless of the codon at the P site and the presence of Ami the rate of tRNA accommodation in the 30S IC formed without IF3 was practically identical [*k*_*app*_(AUG) = 3.97 ± 0.02 s^–1^; *k*_*app*_(UUC) = 3.68 ± 0.04 s^–1^; *k*_*app*_(AUG + Ami) = 3.43 ± 0.02 s^–1^; *k*_*app*_(UUC + Ami) = 3.63 ± 0.02 s^–1^]. As in the case of subunits joining, during tRNA accommodation complex with UUC start codon demonstrated lower signal amplitude that could be restored almost to maximum value (AUG case) by the addition of Ami (Figure 3D). Thus, the absence of IF3 allows the formation of productive 70S ICs that contained initiator tRNA even for erroneous initiation codons. In this context, Ami increased the extent of 70S complexes erroneously formed the UUC start codon.

Our results indicate that Ami has a binary negative impact on initiation: it inhibits formation of canonical ICs and supports emergence of erroneous ICs. Ami decreases the binding strength of the correct mRNA to the 30S IC by a factor of 10. In addition, it greatly reduces (30-fold) the speed of 70S IC formation, thereby significantly suppressing canonical initiation. At the same time, Ami promotes formation of incorrect 30S IC due to increase in the binding affinity of mRNA with an incorrect start codon. Although erroneous complexes are unable to progress along the path of translation initiation due to controlling action of IF3, formation of such complexes requires cellular resources that otherwise could be used for canonical initiation. Analysis of the system lacking IF3 supports a model where the mechanism of translation initiation inhibition is IF3-dependent. The absence of IF3 dramatically reduces the binding affinity of mRNA, however, the resulting complexes rapidly and effectively turn into the 70S IC. Erroneous complexes with an UUC start codon result in moderate decrease in amount of 70S IC formed, which could be readily rescued by addition of Ami, most probably due to an mRNA affinity gain. Thus, Ami increases the amount of incorrect 30S IC converted to the 70S IC that becomes possible in the absence of IF3. Our results highlight controlling function of IF3 as the complete translation initiation system does not allow formation of incorrect 70S IC regardless of Ami presence.

### Amicoumacin A Does Not Interfere With Decoding and Peptide Bond Formation

**FIGURE 4:**
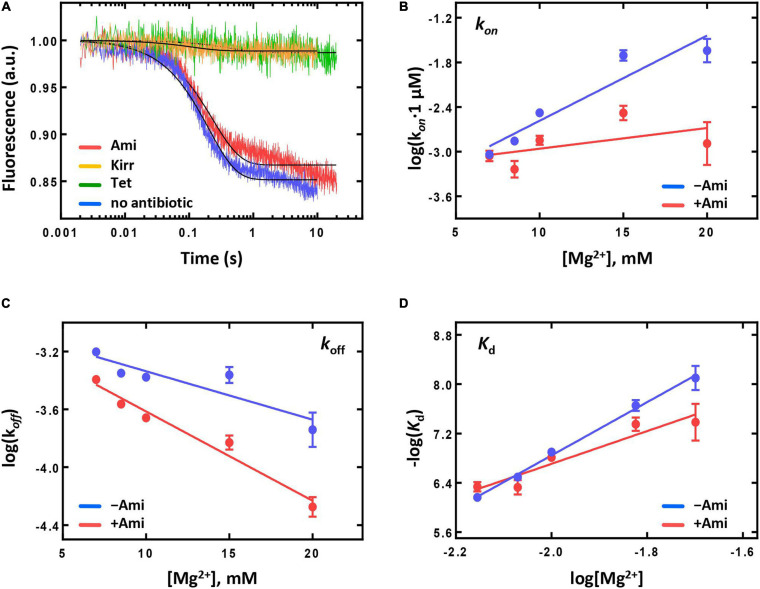
The influence of Ami on the aminoacyl-tRNA binding and the stability of peptidyl-tRNA binding to the A site of the ribosome. **(A)** The pre-steady-state kinetics of the ternary complex EF-Tu⋅GTP⋅Phe-tRNA^Phe^ (2 μM) interaction with the 70S IC (50 nM) containing fMet-tRNA^fMet^(Prf20) at the P site without addition of antibiotic (no antibiotic), in the presence of amicoumacin A (Ami), tetracycline (Tet), and kirromycin (Kirr). The dependence of the *k*_*on*_ **(B)** and *k*_*off*_ **(C)** values on the Mg^2+^ ion concentration obtained for the pretranslocation complex containing deacylated tRNA^fMet^ at the P site and fMet-[^14^C]Val-tRNA^Val^ at the A site. **(D)** The *K*_d_ values calculated from **(B)** to **(C)**.

70S IC formed during translation initiation enters elongation cycle, which consists of decoding, peptide bond formation, and translocation steps (reviewed in Rodnina, 2018). Decoding implies recognition of the mRNA codon at the A site by corresponding aminoacyl-tRNA and its accommodation on the ribosome. The step is completed by the correct positioning of the aminoacyl-tRNA acceptor end in the peptidyl transferase center of the ribosome, leading to the instantaneous peptide bond formation between the P-site fMet and the A-site aminoacyl group. The resulting pretranslocation complex contains deacylated tRNA at the P site and peptidyl-tRNA at the A site of the ribosome. Here, accommodation of Phe-tRNA^Phe^ at the A site of the 70S IC was monitored by the fluorescence change of a fluorescent reporter group proflavine attached to dihydrouridine at position 20 located in the elbow region of initiator tRNA [fMet-tRNA^fMet^(Prf20)] (Figure 4A). The fluorescence change reflects an amendment of Prf20 microenvironment upon approach of Phe-tRNA^Phe^ in the process of its accommodation (Pan et al., 2008). The presence of Ami had impact neither on the amplitude nor on the reaction rate [*k*_*app*_(without Ami) = 4.96 ± 0.05 s^–1^; *k*_*app*_(Ami) = 4.31 ± 0.06 s^–1^]. On the contrary, there was essentially no fluorescence signal change upon application of well-known inhibitors of A-site reactions (Figure 4A). Tetracycline binds to the small ribosomal subunit and sterically violates the recognition of an mRNA codon by an A-site tRNA anticodon (Nguyen et al., 2014). Since tetracycline leads to a dramatic decrease in the rate of tRNA A-site binding (Semenkov YuP et al., 1982), we cannot see accommodation of the A-site tRNA within the time range of the experiment (Figure 4A). Kirromycin does not interfere with the efficient aminoacyl-tRNA binding to the ribosome but suppresses its accommodation in the A site (Rodnina et al., 1995). Thus, presence of antibiotics that inhibit either binding or accommodation of aminoacyl-tRNA does not show the fluorescence change specific for accommodation of tRNA at the A site next to the Prf-labelled initiator tRNA.

Correct accommodation of aminoacyl-tRNA at the A site results in immediate transfer of peptidyl-residue from the P-site tRNA to the A-site tRNA leading to formation of dipeptide fMet-Phe attached to the A-site tRNA. The efficacy of the reaction could be monitored by evaluation of amount of radioactively labelled dipeptide fMet-[^14^C]Phe formed compared to free amino acid [^14^C]Phe. Ami-containing ribosomal complexes were as effective in the reaction of peptide bond formation as intact complexes with the amount of dipeptide fMet-[^14^C]Phe formed equal to 72 ± 7% and 73 ± 8%, respectively. Addition of specific inhibitor of peptide bond formation madumycin II (Osterman et al., 2017) severely impaired formation of fMet-[^14^C]Phe resulting in 33 ± 7%. Thus, neither delivery of Phe-tRNA^Phe^ within ternary complex EF-Tu⋅GTP⋅Phe-tRNA^Phe^ and subsequent accommodation of Phe-tRNA^Phe^, nor the peptide fMet-Phe bond formation were affected by addition of Ami.

As stability of peptidyl-tRNA binding could influence the next step of elongation, translocation, we analyzed the dependence of rate constants (*k*_*on*_ and *k*_*off*_) and dissociation constant (*K*_d_) of peptidyl-tRNA binding at the A site on Mg^2+^ ions concentration (Konevega et al., 2004) (Figures 4B–D). At physiological concentration of Mg^2+^ (7 mM), the presence of the antibiotic did not change *k*_*off*_ and *k*_*on*_ values and had only moderate impact on the affinity of peptidyl-tRNA to the A site [*K*_d_(without Ami) = 688 ± 88 nM; *K*_d_(Ami) = 461 ± 81 nM]. At elevated Mg^2+^ concentration (up to 20 mM), the rate of the peptidyl-tRNA dissociation decreased three times, the rate of association decreased 17 times, and the affinity difference increased five-fold. The result indicates that Ami reduces the amount of Mg^2+^ ions required for dissociation and association, as well as for stabilization of peptidyl-tRNA at the A site. Nevertheless, Ami does not change the stability of peptidyl-tRNA binding at the A site at physiological conditions and does not affect the free energy (ΔG^0^) of binding calculated directly from the *K*_d_ values according to the equation ΔG^0^ = -RTln(*K*_d_) [ΔG^0^ (without Ami) = −8.74 ± 0.08 kcal/mol; ΔG^0^ (Ami) = −8.98 ± 0.11 kcal/mol] (Konevega et al., 2004). Thus, the pretranslocation complexes containing Ami can enter translocation with the same free energy of the ground state as complexes without the antibiotic.

### Amicoumacin A Decreases the Amount of Active Ribosomes

During translocation displacement of mRNA and tRNAs occurs as synchronous and concerted large-scale movement in the intersubunit space of the ribosome (Holtkamp et al., 2014). The deacylated tRNA moves from the P to the E site and rapidly dissociates from the ribosome. Relocation of the peptidyl-tRNA from the A to the P site is accompanied by mRNA shift ensuring positioning of a new codon at the A site in order to bind the corresponding aminoacyl-tRNA at the next elongation cycle (Rodnina, 2018). It was previously shown that addition of some inhibitors of translocation uncoupled movement of acceptor stem of tRNA and mRNA (Holtkamp et al., 2014). Here we set to compare movement of acceptor stem and elbow region of tRNA upon addition of Ami. To study the antibiotic influence on a single-round translocation, we used pretranslocation complexes containing deacylated tRNA^fMet^ at the P site and fluorescently labelled fMet-Phe-tRNA^Phe^ at the A site of the ribosome. To characterize movement of the acceptor end of tRNA from the A to the P site we have used Met-Phe-tRNA^Phe^ labelled with Bodipy FL at methionine moiety (BPY-Met-Phe-tRNAPhe) (Holtkamp et al., 2014). To monitor displacement of central part of tRNA upon translocation, we have used proflavine attached to dihydrouridine at position 16 and/or 17 located in the elbow region of fMet-Phe-tRNA^Phe^ [fMet-Phe-tRNA^Phe^(Prf16/17)] (Savelsbergh et al., 2000).

**FIGURE 5:**
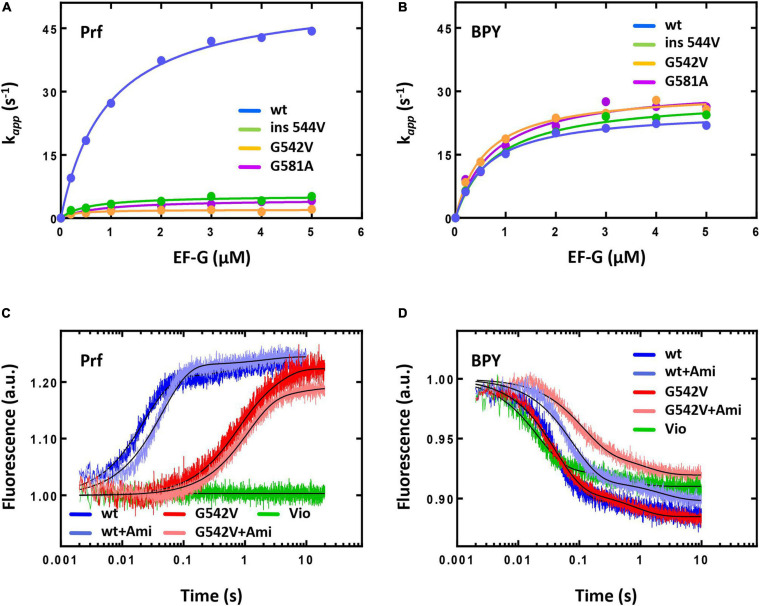
The pre-steady state kinetics of translocation catalyzed by intact or mutant forms of EF-G. The dependence of the fast translocation phase rate on the concentration of EF-G monitored by the fluorescence change of fMet-Phe-tRNA^Phe^(Prf16/17) **(A)** or BPY-Met-Phe-tRNA^Phe^ **(B)**. The time courses of single round translocation (at saturating concentration of EF-G (5 μM) monitored by the fluorescence change of fMet-Phe-tRNA^Phe^(Prf16/17) **(C)** or BPY-Met-Phe-tRNA^Phe^ **(D)**. Viomycin (Vio). Error bars (s.d.) in **(A,B)** were obtained from at least two independent experiments with 5–7 technical replicates each, however, do not exceed the size of symbols.

The kinetics of translocation was monitored by the fluorescence change upon addition of EF-G to fluorescently labelled pretranslocation complexes. Upon addition of saturating concentration of EF-G (4 μM) the displacement of peptidyl-tRNA occurred with the rate constant of *k* = 42 s^–1^ for complexes containing reporter in the tRNA elbow region (Prf) and *k* = 22 s^–1^ for complexes containing reporter at the acceptor end of tRNA (Figures 5A,B). Interestingly, previous studies demonstrated *k* values close to 30 s^–1^ in cases of both reporters (Holtkamp et al., 2014), that could be associated with different variants of EF-G used in this study (His-tag at the *N*-terminal end of the protein) and earlier (*C*-terminal His-tag).

The presence of Ami decreased the rate of peptidyl-tRNA displacement from the A to the P site of the ribosome by two-fold when monitored by reporters at the elbow and the acceptor end region of the tRNA. Wherein the antibiotic did not affect the fluorescence signal amplitude and the ratio of fast and slow translocation phases [for the fast phase Prf: *k*(without Ami) = 43.6 ± 0.4 s^–1^; *k*(Ami) = 22.3 ± 0.2 s^–1^; BPY: *k*(without Ami) = 21.7 ± 0.2 s^–1^; *k*(Ami) = 12.6 ± 0.1 s^–1^; in both cases amplitude of the fast phase A > 85%] (Figures 5C,D). Since additional Mg^2+^ ions stabilize the ribosome structure and could enhance the interaction of the antibiotic with the mRNA and 16S rRNA, we examined the kinetics of translocation at the increased Mg^2+^ ion concentration (20 mM). Even in this case the presence of the antibiotic diminished the rate of the peptidyl-tRNA displacement by three-fold both with labels at the elbow and acceptor end region (Supplementary Figure S1). This effect was principally different from that known for other antibiotics acting on translocation. For example, viomycin binds between two ribosomal subunits, blocks mRNA-tRNA complex displacement and 30S subunit rotation (Holtkamp et al., 2014). At single round translocation, viomycin impeded the tRNA movement at the elbow region and increased the reaction rate at its acceptor end [BPY: *k* (Vio) = 47.2 ± 0.6 s^–1^] (Figures 5C,D).

**FIGURE 6:**
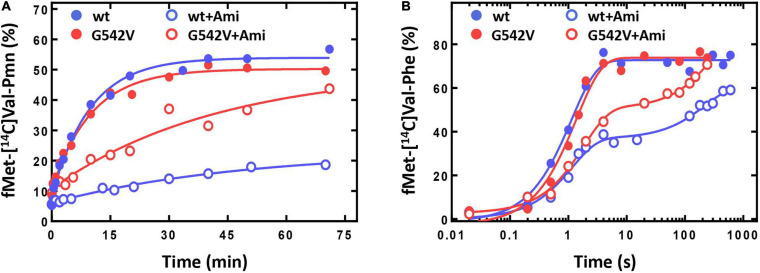
The influence of Ami on the multiple turnover translocation catalyzed by intact or G542V EF-G. **(A)** The time courses of fMet-[^14^C]Val-puromycin formation. **(B)** The time courses of fMet-[^14^C]Val-Phe tripeptide formation.

The antibiotic affected not only a single round of translocation, but also significantly altered the involvement of the ribosome and EF-G in the multiple turnover translocation. To test this, we used puromycin (Pmn) that binds at the A site of the post-translocated ribosome, intercepts a nascent polypeptide from the peptidyl-tRNA, and dissociates (Savelsbergh et al., 2000). In fMet-[^14^C]Val-Pmn formation assay we tested catalytic concentrations of different variants of EF-G (Supplementary Figure S3). The presence of the antibiotic decreased the translocation rate five times [*k*(without Ami) = 0.103 ± 0.010 min^−1^; *k*(Ami) = 0.020 ± 0.005 min^−1^], and reduced the amount of peptidyl-tRNA translocated from the A to the P site of the ribosome three times (Figure 6A).

This effect could be associated with the change of EF-G engagement in the translocation or decrease of the ribosomal complex activity after a single round of translocation in the presence of Ami. To test this assumption, we performed the analysis of the tripeptide fMet-Val-Phe formation. EF-G and ternary complex EF-Tu⋅GTP⋅Phe-tRNA^Phe^ were added to the pretranslocation complexes containing deacylated tRNA^fMet^ at the P site and fMet-[^14^C]Val-tRNA^Val^ at the A site, followed by the sampling at certain time points. Tripeptide formation in the presence of the antibiotic revealed a two-phase curve in contrast to the one-phase curve obtained for intact complexes (Figure 6B). Ami did not perturb the fast phase rate, similar rates of tripeptide formation were obtained in the absence of the antibiotic *k*(without Ami) = 0.9 ± 0.1 s^–1^; *k*(Ami) = 0.9 ± 0.1 s^–1^. However, the fast phase reached only a half (35% of 75%) of the formed tripeptide amplitude curve, and the final level of the curve was lower even after extended incubation (the maximum amplitude was 61%). This indicates that a large portion of 70S complexes were impaired for translation.

Ami slows down movement of the peptidyl-tRNA from the A to the P site of the ribosome without influencing the conformational dynamics of tRNA displacement, as the rate of translocation monitored at different parts of the tRNA decreased by the same factor. However, the results of multiple turnover translocation and the tripeptide formation designate that Ami significantly decreases the portion of the ribosomes involved into the next elongation cycle.

### Mechanism of Resistance to Amicoumacin A

Naturally occurring mutations can modify certain components of the translational system enabling them to neutralize the inhibitory effects of antibiotics. Investigation of such elements contributes to elucidating not only the mechanism of antibiotic resistance, but also the molecular mode of antibiotics action. Interestingly, all detected modifications of EF-G (substitutions G581A, G542V, and insertion ins544V), providing Ami resistance, are located more than 16 Å away from the binding site of the antibiotic (Polikanov et al., 2014), being, at the same time, at close proximity to the tRNA-mRNA complex on the ribosome (Gao et al., 2009). The modifications belong to structural elements that are spatially close located to two conserved loops I and II [β2–β3 (508–514 aa) and β5-α_B_ (584–590 aa)] (Figure 1). These loops contact mRNA and the anticodon region of the peptidyl-tRNA at the P site of the posttranslocation complex and presumably the mRNA-tRNA duplex at the A site of the pretranslocation complex (Gao et al., 2009; Tourigny et al., 2013; Liu et al., 2014). Thus, they are expected to accompany the mRNA-tRNA duplex during translocation from the A to the P site of the ribosome. At the same time, biochemical properties and performance *in vitro* of the EF-G variants were not assessed.

First, we checked the protein stability due to the amino acid substitutions by Nano differential scanning fluorimetry method (Wen et al., 2020). Our results show that all studied EF-G variants had very similar profiles of temperature-driven protein unfolding. The calculated thermal transition temperature was 60.8°C for the *wt* EF-G while variants of EF-G deviated less than 2°C (Supplementary Figure S2), suggesting that amino acid alterations under study did not affect global conformational stability of EF-G.

Second, we studied the translocation reaction for all modified variants of EF-G showing that they efficiently catalyze multiple turnover translocation. According to the puromycin reaction assay of fMet-[^14^C]Phe-Pmn formation the rate constants were *k* (EF-G wt) = 0.09 ± 0.02 min^−1^, *k* (EF-G G542V) = 0.10 ± 0.02 min^−1^, *k* (EF-G ins544V) = 0.09 ± 0.01 min^−1^, *k* (EF-G G581A) = 0.07 ± 0.01 min^−1^ at 3 nM EF-G (Supplementary Figure S3). However, single round translocation was greatly affected by the mutations. The rate of the fast translocation phase of peptidyl-tRNA labelled at the elbow region decreased 10–12 times upon addition of EF-G with G581A (*k* = 4.1 ± 0.1 s^–1^) or ins544V (*k* = 5.2 ± 0.2 s^–1^) and 25 times with G542V (*k* = 2.1 ± 0.1 s^–1^). The ratio of translocation phases also changed with the amplitude of the fast phase decreased to 60%, increasing the contribution of the slow phase for all mutant forms of EF-G. Interestingly, modified variants of EF-G did not affect the displacement rate of the peptidyl-tRNA labelled at the acceptor end region (G581A *k* = 26.3 ± 0.4 s^–1^, ins544V *k* = 24.4 ± 0.3 s^–1^, G542V *k* = 25.7 ± 0.3 s^–1^), as well as the ratio of the translocation phases (Figures 5A,B). Thus, during single round of translocation mutant EF-G exhibits strong alteration of the peptidyl-tRNA conformational dynamics at its displacement from the A to the P site of the ribosome.

To study the mechanism of Ami resistance we used EF-G G542V, as all mutant variants of EF-G had similar phenotype and this variant of the protein demonstrated the most pronounced effects on translocation. In the presence of Ami single round translocation catalyzed by EF-G G542V showed that the rate of the peptidyl-tRNA displacement with both labels, at the elbow and acceptor end region, was decreased about 2–3 times, slightly affecting the ratio of translocation phases [Prf: *k*(without Ami) = 1.65 ± 0.07 s^–1^; *k*(Ami) = 0.92 ± 0.02 s^–1^; BPY: *k*(without Ami) = 24.5 ± 0.2 s^–1^; *k*(Ami) = 8.9 ± 0.2 s^–1^] (Figures 5C,D). In other words, EF-G variant under study alters the peptidyl-tRNA conformational dynamics during translocation, whereas Ami slows down tRNA movement not affecting its conformation any further.

The analysis of Ami-inhibited multiple turnover translocation stimulated by EF-G G542V showed five times decrease of the reaction rate [*k*(without Ami) = 0.099 ± 0.009 s^–1^; *k*(Ami) = 0.024 ± 0.009 s^–1^]. The same extent of inhibition by Ami was observed for intact EF-G. Interestingly, despite of equal rate reduction the amount of translocated peptidyl-tRNA was different. Modified variant of EF-G ensured maximal amplitude of the reaction, whereas the amount of translocated tRNA in the presence of native EF-G was reduced by a factor of three (Figure 6A).

The rates of tripeptide fMet-Val-Phe formation supported by EF-G variants were similar [*k*(EF-G) = 0.73 ± 0.08 s^–1^; *k*(EF-G G542V) = 0.55 ± 0.06 s^–1^] (Figure 6B). Addition of Ami in both cases led to transformation of a single-exponential reaction into a two-phase curve. Despite the delay caused by the second slow phase the amount of tripeptide formed with EF-G G542V reached maximum, while native variant of EF-G could ensure only 85% of tripeptide.

To sum up, at a single round translocation Ami-resistant variant of EF-G G542V changes the conformational dynamics and the rate of the peptidyl-tRNA displacement from the A to the P site of the ribosome. However, in the presence of Ami the participation of modified variant of EF-G provides the transition of larger portion of the ribosomes to the next elongation cycle in comparison to the action of intact form of EF-G. It is possible that the changes in translocation induced by EF-G G542V maintain the activity of the Ami-bound ribosomes.

## Discussion

Our study shows that the main mechanism of translation inhibition by Ami can be assigned to the initiation and translocation phases. At the initiation step, Ami as well as another antibiotic Ede alter mRNA binding to the 30S subunit. While Ede severely disturbs 30S IC formation leading to complete inability to produce 70S IC, Ami performs more delicate. Our data indicate that Ami perturbs the AUG decoding step in two ways, slightly reduces the mRNA affinity for the canonical AUG codon but greatly increases that for 30S complexes with the erroneous initiation codon UUC (Figure 2). Among all three initiation factors, IF3 actively promotes initiation codon decoding, enhancing it for AUG and repressing others (La Teana et al., 1993). Additionally, the progression of 30S IC towards elongation is kinetically checked by the factor (Milon et al., 2008). Thus, IF3 performs as a fidelity factor. The Ami binding site overlaps with that of IF3 (Fabbretti et al., 2007; Julián et al., 2011; Polikanov et al., 2014; Prokhorova et al., 2016), suggesting that the mechanism of Ami-dependent perturbance may be coupled to IF3 fidelity functions. We observe that Ami greatly reduces the velocity of 70S IC formation for complexes containing a model mRNA programmed with an AUG initiation codon. Interestingly, a similar reduction was observed in previous studies if the mRNA contained an extended Shine-Dalgarno sequence or non-canonical initiation codons (Grigoriadou et al., 2007; Milon et al., 2008). Alterations of the mRNA-16S rRNA interaction architecture induced by a long Shine-Dalgarno anti-Shine-Dalgarno duplex or codon-anticodon base pairing upon non-canonical initiation are sensed by IF3 and define the speed at which the ribosome enters translation elongation. It seems that binding of Ami could also be detected by IF3. Indeed, omission of IF3 from the reaction fully abrogated any difference between the presence or absence of the antibiotic (Figure 3).

Our data also suggest that Ami blocks IF3-dependent rejection of non-canonical initiation codons (Figures 2, 3). We observed that Ami increases the amount of 30S IC formed with the UUC initiation codon, suggesting that the antibiotic increases erroneous translation initiation. However, the erroneous 30S ICs are not capable of transit towards elongation as subunit joining and tRNA accommodations are impaired. Importantly, this observation strengthens the dual relation between IF3 and Ami during translation initiation. A plausible scenario would entail the antibiotic locking IF3 in an intermediate conformation that promotes codon–anticodon interaction but fails to dissociate.

At the translocation step, Ami reduces the rate of peptidyl-tRNA movement and, more importantly, decreases the amount of the ribosomes able to be involved in the next elongation cycle. This effect could be associated with the binding site of the antibiotic: Ami is located between the head and the platform domains of the 30S subunit and could attach 5′-end of the mRNA to the conservative nucleotides of both domains (Jenner et al., 2010; Polikanov et al., 2014). Our data fully support this assumption by demonstration that Ami stabilizes mRNA at the 30S IC. The antibiotic slows down but allows the mRNA-tRNA complex movement meaning that initial rotation of the 30S subunit is not impaired. At the same time, the loss of the ribosome functional activity implies blockage at some later steps. There is a probability that the reverse rotation of the head and the body domains of the 30S might be hindered due to their stabilization to each other and the mRNA retention, however, one still needs additional experimental evidence to support this speculation.

The variant of EF-G with substitution G542V leads to a 25-fold decrease in the rate of movement of the elbow region of peptidyl-tRNA with additional amplification of the effect by the presence of Ami. Nevertheless, this EF-G variant retains the functional activity of the ribosomes during their transition to the next round of elongation and provides resistance to Ami. Interestingly, all point amino acid variations of EF-G impairing single round translocation and conferring resistance to Ami contact neither the antibiotic, nor the tRNA-mRNA duplex: G581A is located at the β5-strand edge (Figure 1), G542V and the insertion ins544V reside in the loop β4-αA (538–548 aa) of EF-G. These areas belong to the structural elements of EF-G that were barely studied as their impact on translocation has never been shown before. At the same time β5-strand and β4-αA loop are spatially very close to conservative loops β2–β3 (508–514 aa) and β5-α_B_ (584–590 aa), also known as loops I and II, which contact mRNA and the anticodon region of the peptidyl-tRNA at the P site of the posttranslocation complex and supposedly mRNA-tRNA duplex at the A site of the pretranslocation complex, accompanying it during translocation from the A to the P site of the ribosome (Gao et al., 2009; Tourigny et al., 2013; Liu et al., 2014).

The phenotype of mutations in the loops I and II is well described, in particular, the replacement of His583 (loop II) slows down the rate of peptidyl-tRNA movement at the elbow region without affecting translocation at the acceptor end of the tRNA (Savelsbergh et al., 2000; Holtkamp et al., 2014) similarly to the variants of EF-G described in our work. The subsequent study of loops I and II showed that amino acid substitutions increased the rate of forward 30S subunit head and body domains rotation with the accompanying movement of tRNAs to the corresponding sites and decreased the rate of back rotation of domains and the mistiming of their movement with the tRNA displacement (Liu et al., 2014; Zhang et al., 2015; Peng et al., 2019). Although EF-G sequence alterations studied in this work do not belong to the loops I or II, they have comparable effect on the peptidyl-tRNA displacement and on the ribosome conformational dynamics. The use of EF-G G542V significantly enhanced the portion of the ribosomes able to proceed to the next translation cycle despite inhibiting action of Ami. Evidently, the retention of the ribosome activity is associated with the alteration of ribosome conformation caused by the participation of modified variant of EF-G in translocation. The replacement of glycine for valine or insertion of additional valine led to increase of the EF-G hydrophobic surface contacting with 16S rRNA, which could be a reason for attenuation of contacts between the ribosome and the factor, reducing the Ami stabilizing effect. The induced changes could be a lot like transformations caused by EF-G with single amino acid substitutions in loops I and II, as retardation of 30S subunit head and body back rotation and its mistiming with tRNA displacement would compensate the Ami stabilizing effect. This observation underlines, that loop β4-α_A_ and β5-strand of EF-G could be as important for translocation as thoroughly studied loops I and II, although the former do not have direct contacts to the mRNA-tRNA complex. Thus, our study has identified elements of EF-G that play significant role in translocation, suggesting functional role of the domain IV structural elements that was not shown earlier.

Ami shares its binding site on the ribosome with several antibiotics, however, their mechanisms of protein synthesis inhibition are quite distinct. Edeine severely suppresses the translation initiation by interfering with the initiator tRNA binding to the P site (Pioletti et al., 2001; Dinos et al., 2004). Inhibition of translation by kasugamycin is intricately linked to the sequence of the mRNA upstream of the start initiation codon (Chin et al., 1993; Schluenzen et al., 2006; Schuwirth et al., 2006; Vázquez-Laslop and Mankin, 2018). Pactamycin acts as a tRNA-specific antibiotic and prevents movement of certain tRNAs upon translocation (Dinos et al., 2004). Ami demonstrates pleiotropic effects on the translation cycle on the ribosome. During initiation Ami distorts the mRNA binding to the 30S IC, hence decreasing the rate of initiator tRNA accommodation and the 50S subunit association. At the translocation step, the antibiotic decreases the rate of the mRNA-tRNA complex movement. However, the most intriguing effect of Ami is its ability to alter the ribosome functional activity in a way that only a portion of the ribosomes proceeds to the next elongation cycle. Modified EF-G variants, exhibiting resistance to Ami, cope with particularly this feature. The main mechanism of action of Ami, which we propose, is based both on our data and on the phenotypic similarity of modified variants of EF-G presented in this study (G542V, G581A, and ins544V) and earlier (substitutions in loops I and II). Namely, the mRNA is stabilized by Ami that interacts with the head and body of the 30S IC, allowing forward rotation and strongly impairing reverse rotation of the head and the body of the 30S IC, thus fixing the ribosome in “non-productive” conformation. More detailed study of the dynamics of the ribosomal complexes in the presence of Ami is necessary to confirm our assumption. However, we can claim that Ami belongs to the very promising group of antibiotics aiming at changing the conformational dynamics of the ribosome.

## Data Availability Statement

The raw data supporting the conclusions of this article will be made available by the authors, without undue reservation.

## Author Contributions

EM, DV, and IO carried out the experiments. IO and PK provided materials. EM, DV, ON, and AP analyzed the data and created the pictures. EM, DV, and AK designed the experiments. OD, IO, PS, PM, AP, and AK conceived and curated the research and interpreted the results. EM, DV, AP, PM, and AK wrote the manuscript. All authors contributed to the article and approved the submitted version.

## Conflict of Interest

AK is a founder of the company NanoTemper Technologies Rus (St. Petersburg, Russia), which provides services and devices based on MST and nanoDSF and represents NanoTemper Technologies GmbH (Germany). The remaining authors declare that the research was conducted in the absence of any commercial or financial relationships that could be construed as a potential conflict of interest.

## Abbreviations

Ami: amicoumacin A
DSF: differential scanning fluorimetry
Ede: edeine
IC: initiation complex
Ksg: kasugamycin
MST: microscale thermophoresis
Pmn: puromycin.
