# Supplementary material for: Multifaceted Mechanism of Amicoumacin A Inhibition of Bacterial Translation
Source: Front Microbiol. 2021 Feb 12;12:618857. doi: 10.3389/fmicb.2021.618857 (PMC7907450; doi:10.3389/fmicb.2021.618857)
Supplement: Supplementary file 1 [file Data_Sheet_1.docx]

Supplementary Material

Multifaceted mechanism of Amicoumacin A Inhibition of Bacterial Translation

**Elena M. Maksimova^1^, Daria S. Vinogradova^1,2^, Ilya A. Osterman^3,4^, Pavel S. Kasatsky^1^,
Oleg S. Nikonov^5^, Pohl Milón^6^, Olga A. Dontsova^3,4,7,8^, Petr V. Sergiev^3,4,7,9^, Alena Paleskava^1*^, Andrey L. Konevega^1,10*^**

^1^Petersburg Nuclear Physics Institute named by B.P. Konstantinov, NRC "Kurchatov Institute", Gatchina, Russia

^2^NanoTemper Technologies Rus, Saint Petersburg, Russia

^3^Center of Life Sciences, Skolkovo Institute of Science and Technology, Skolkovo, Moscow Region, Russia

^4^Department of Chemistry, Lomonosov Moscow State University, Moscow, Russia

^5^Institute of Protein Research, Russian Academy of Science, Pushchino, Russia

^6^Centre for Research and Innovation, Faculty of Health Sciences, Universidad Peruana de Ciencias Aplicadas (UPC), Lima, Peru

^7^A.N. Belozersky Institute of Physico-Chemical Biology, Lomonosov Moscow State University, Moscow, Russia

^8^Shemyakin-Ovchinnikov Institute of Bioorganic Chemistry, Moscow, Russia

^9^Institute of Functional Genomics, Lomonosov Moscow State University, Moscow, Russia

^10^National Research Centre "Kurchatov Institute", Moscow, Russia

*** Correspondence:**Andrey L. Konevega
konevega_al@pnpi.nrcki.ru

Alena Paleskava

polesskova_ev@pnpi.nrcki.ru

**This file includes:**

1. Supplementary Figures S1 to S3 with legends

**I. SUPPLEMENTARY FIGURES**


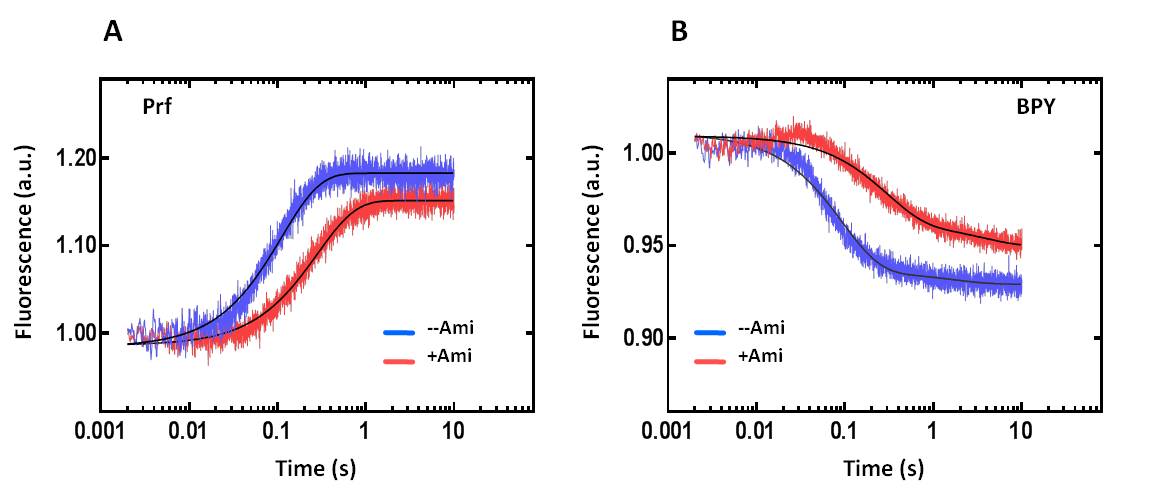


**Figure S1. The pre-steady state kinetics of translocation at the increased Mg^2+^ ion concentration.** The experiments were performed for the pretranslocation complexes (60-80 nM) containing deacylated tRNA^fMet^ at the P site, fMet-Phe-tRNA^Phe^(Prf16/17) **(A)** or BPY-Met-Phe-tRNA^Phe^ **(B)** at the A site at a saturating concentration of EF-G (5 μM) in buffer TAKM_20_. The calculated values of the fast translocation phase rate constants from the fluorescence change of the Prf reporter were *k*(without Ami) = 8.97 ± 0.06 s^–1^; *k*(Ami) = 3.52 ± 0.02 s^-1^; the BPY reporter: *k*(without Ami) = 10.8 ± 0.1 s^-1^; *k*(Ami) = 3.42 ± 0.07 s^-1^


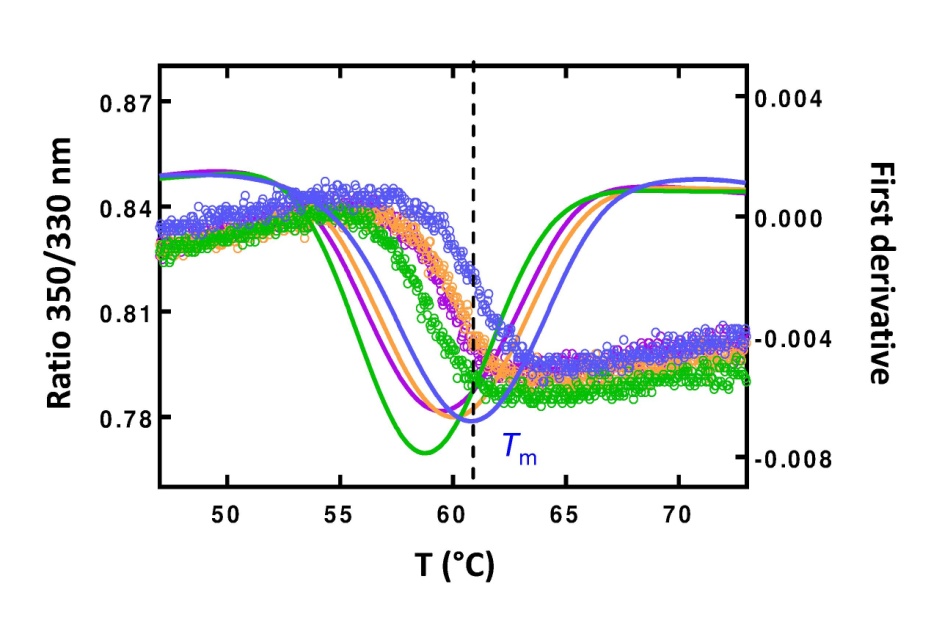


**Figure S2. The thermal stability of the intact and mutant EF-G forms.** The graph shows T_m_ and the first derivative curves for the intact and mutant forms of EF-G. The presented fluorescence changes (○) and the first derivative (─) for the intact form (indicated in blue) EF-G or EF-G containing G542V (orange), ins544V (green) or G581A (purple) correspond to T_m_(EF-G wt) = 60.8°C, T_m_(EF-G G542V) = 60.0°C, T_m_(EF-G ins544V) = 58.8°C, T_m_(EF-G G581A) = 59.5°C. GTP-bound forms of EF-G were used for the experiments.


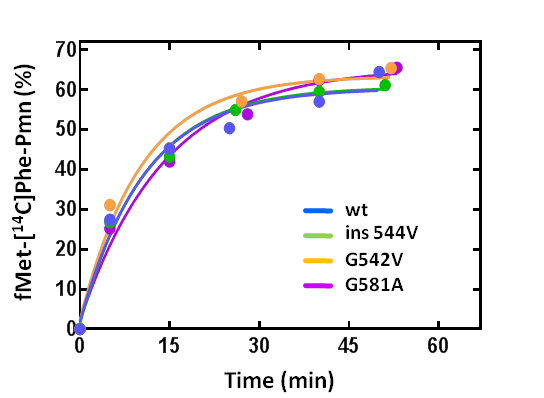


**Figure S3. The implication of the intact and mutant EF-G forms in the multiple turnover translocation.** The time courses of fMet-[^14^C]Phe-puromycin formation. The calculated values of the rate constants are *k*(EF-G wt) = 0.09 ± 0.02 min^-1^, *k*(EF-G G542V) = 0.10 ± 0.02 min^-1^, *k*(EF-G ins544V) = 0.09 ± 0.01 min^-1^, *k*(EF-G G581A) = 0.07 ± 0.01 min^-1^.
